# Dystrophin R16/17 protein therapy restores sarcolemmal nNOS *in trans* and improves muscle perfusion and function

**DOI:** 10.1186/s10020-019-0101-6

**Published:** 2019-07-02

**Authors:** Junling Zhao, Hsiao Tung Yang, Lakmini Wasala, Keqing Zhang, Yongping Yue, Dongsheng Duan, Yi Lai

**Affiliations:** 1 Department of Molecular Microbiology and Immunology, School of Medicine, University of Missouri, Medical Sciences Building, One Hospital Drive, Columbia, MO 65212 USA; 2 Department of Biomedical Sciences, College of Veterinary Medicine, University of Missouri, Columbia, MO 65212 USA; 3 Department of Neurology, School of Medicine, University of Missouri, Columbia, MO 65212 USA; 4 Department of Bioengineering, University of Missouri, Columbia, MO 65212 USA

**Keywords:** Dystrophin R16/17, Blood perfusion, Protein therapy, Sarcolemmal nNOS, Functional ischemia, Dystrophinopathy

## Abstract

**Background:**

Delocalization of neuronal nitric oxide synthase (nNOS) from the sarcolemma leads to functional muscle ischemia. This contributes to the pathogenesis in cachexia, aging and muscular dystrophy. Mutations in the gene encoding dystrophin result in Duchenne muscular dystrophy (DMD) and Becker muscular dystrophy (BMD). In many BMD patients and DMD patients that have been converted to BMD by gene therapy, sarcolemmal nNOS is missing due to the lack of dystrophin nNOS-binding domain.

**Methods:**

Dystrophin spectrin-like repeats 16 and 17 (R16/17) is the sarcolemmal nNOS localization domain. Here we explored whether R16/17 protein therapy can restore nNOS to the sarcolemma and prevent functional ischemia in transgenic mice which expressed an R16/17-deleted human micro-dystrophin gene in the dystrophic muscle. The palmitoylated R16/17.GFP fusion protein was conjugated to various cell-penetrating peptides and produced in the baculovirus-insect cell system. The best fusion protein was delivered to the transgenic mice and functional muscle ischemia was quantified.

**Results:**

Among five candidate cell-penetrating peptides, the mutant HIV trans-acting activator of transcription (TAT) protein transduction domain (mTAT) was the best in transferring the R16/17.GFP protein to the muscle. Systemic delivery of the mTAT.R16/17.GFP protein to micro-dystrophin transgenic mice successfully restored sarcolemmal nNOS without inducing T cell infiltration. More importantly, R16/17 protein therapy effectively prevented treadmill challenge-induced force loss and improved muscle perfusion during contraction.

**Conclusions:**

Our results suggest that R16/17 protein delivery is a highly promising therapy for muscle diseases involving sarcolemmal nNOS delocalizaton.

**Electronic supplementary material:**

The online version of this article (10.1186/s10020-019-0101-6) contains supplementary material, which is available to authorized users.

## Background

Neuronal nitric oxide synthase (nNOS) is an important enzyme that catalyzes the synthesis of a signaling molecule, nitric oxide (NO). NO has an extremely short half-life and cannot diffuse to distant targets. Proximal localization of nNOS to its targets is thus critical for NO-mediated cellular activities. In skeletal muscle, nNOS is predominantly confined to the sarcolemma (Brenman et al. 1995; Stamler and Meissner 2001), and is responsible for the majority of NOS activity in skeletal muscle (Stamler and Meissner 2001). It is well-established that sarcolemmal nNOS plays important roles in muscle function (Stamler and Meissner 2001), including blood perfusion (Thomas et al. 1998, 2003), glucose metabolism (Balon and Nadler 1997; Roberts et al. 1997; Roy et al. 1998; Wehling-Henricks et al. 2009), oxidative stress (Tews 2001; Khan et al. 2004), muscle contractility (Percival et al. 2008; Li et al. 2011), muscle satellite cell activation and muscle repair (Wozniak and Anderson 2009; Buono et al. 2012; Yin et al. 2013; Dumont and Rudnicki 2016), mitochondria biogenesis (Schild et al. 2006; Wadley et al. 2007; Tengan et al. 2012; De Palma et al. 2014), muscle mass (Suzuki et al. 2007; Pietri-Rouxel et al. 2010; Meinen et al. 2012; Lawler et al. 2014), and muscle fatigue (Kobayashi et al. 2008; Percival et al. 2010; Percival 2011; Meinen et al. 2012). Of particular interest is the role nNOS plays in muscle blood flow regulation. During exercise, sympathetic nerve activity leads to vasoconstriction. Simultaneously, exercise dramatically elevates cytosolic Ca^2+^ concentration, and thereby promotes the interaction of nNOS with calmodulin (Stamler and Meissner 2001). Binding of calmodulin to nNOS activates nNOS and increases NO production (Zhou and Zhu 2009; Tejero et al. 2010). Sarcolemmal localization of nNOS allows immediate release of NO to the surrounding vasculature. Consequently, NO-induced vasodilation maintains sufficient blood perfusion in the contracting muscle. Loss of sarcolemmal nNOS impairs NO-mediated vasodilation and leads to functional ischemia. Clinically, patients lacking sarcolemmal nNOS experience muscle fatigue, pain, cramp and eventually histological lesions and force reduction (Thomas et al. 1998; Sander et al. 2000; Kobayashi et al. 2008; Li et al. 2010, 2011; Martin et al. 2012; Kodippili et al. 2018).

Sarcolemmal nNOS localization is disrupted in cachexia, aging-related muscle atrophy (Acharyya et al. 2005; Samengo et al. 2012), and a variety of neuromuscular disorders (Finanger Hedderick et al. 2011). Duchenne muscular dystrophy (DMD) (Brenman et al. 1995; Chang et al. 1996) and Becker muscular dystrophy (BMD) (Chao et al. 1996; Torelli et al. 2004) are two classic examples of sarcolemmal nNOS delocalization. DMD and BMD are a group of the muscle wasting diseases caused by mutations in the dystrophin gene. In DMD, gene mutations result in a complete loss of dystrophin. BMD patients usually carry in-frame deletions and hence express a truncated dystrophin protein and show a relatively mild clinical phenotype.

In the mid 1990s, the Bredt lab demonstrated that dystrophin is responsible for sarcolemmal localization of nNOS (Brenman et al. 1995). We previously identified dystrophin spectrin-like repeats 16 and 17 (R16/17) as the nNOS-binding domain for dystrophin to tether nNOS to the sarcolemma (Lai et al. 2009). R16/17 is encoded by exons 42 to 46 of the dystrophin gene. This overlaps with exons 43–55, a region that is most frequently deleted in DMD and BMD patients (White and den Dunnen 2006). While an effective gene therapy for DMD may require expression of a functional dystrophin protein, restoration of sarcolemmal nNOS alone should substantially ameliorate symptoms associated with functional ischemia in BMD patients. DNA-level CRISPR editing and RNA-level exon-skipping have been actively pursued to convert DMD to BMD through production of a truncated dystrophin protein. Several clinical trials have also been started in DMD patients to deliver micro-dystrophins that do not carry the nNOS binding domain (Duan 2018). However, for DMD patients that already have deletion mutations in exons 42–46, these therapies will remain incomplete because they cannot restore sarcolemmal nNOS. A strategy that can restore sarcolemmal nNOS would greatly benefit these patients.

ΔR4-R23/ΔC (ΔR4) micro-dystrophin has been shown to improve muscle function and histology of the dystrophic muscle (Harper et al. 2002; Gregorevic et al. 2004; Liu et al. 2005; Gregorevic et al. 2006). However, due to lack of the R16/17 nNOS-binding domain, ΔR4 micro-dystrophin cannot anchor nNOS to the sarcolemma (Yue et al. 2006). Here, we used ΔR4 mice to mimic the condition of BMD or DMD receiving exon skipping or gene editing therapy, where truncated dystrophins cannot restore sarcolemmal nNOS.

Protein therapy has been widely used to treat human diseases (Crunkhorn 2013; Gorzelany and de Souza 2013). In this study, we explored whether delivery of a recombinant R16/17 protein could restore sarcolemmal nNOS and attenuate functional ischemia in ∆R4 mice. Specifically, we identified a cell penetrating peptide (CPP) that can mediate effective protein delivery to the muscle. We then fused this CPP to a palmitoylated (Pal) R16/17-GFP protein and systematically delivered the purified fusion protein to ∆R4 mice for 7 weeks. R16/17 protein delivery successfully recovered sarcolemmal nNOS. More importantly, it significantly improved blood perfusion in the contracting muscle and prevented functional ischemia-induced force loss. Our results lay the foundation for further development of R16/17 protein transfer as a novel therapy for sarcolemmal nNOS-deficient muscle diseases.

## Methods

### Baculoviral plasmid construction

**Fig. 1:**
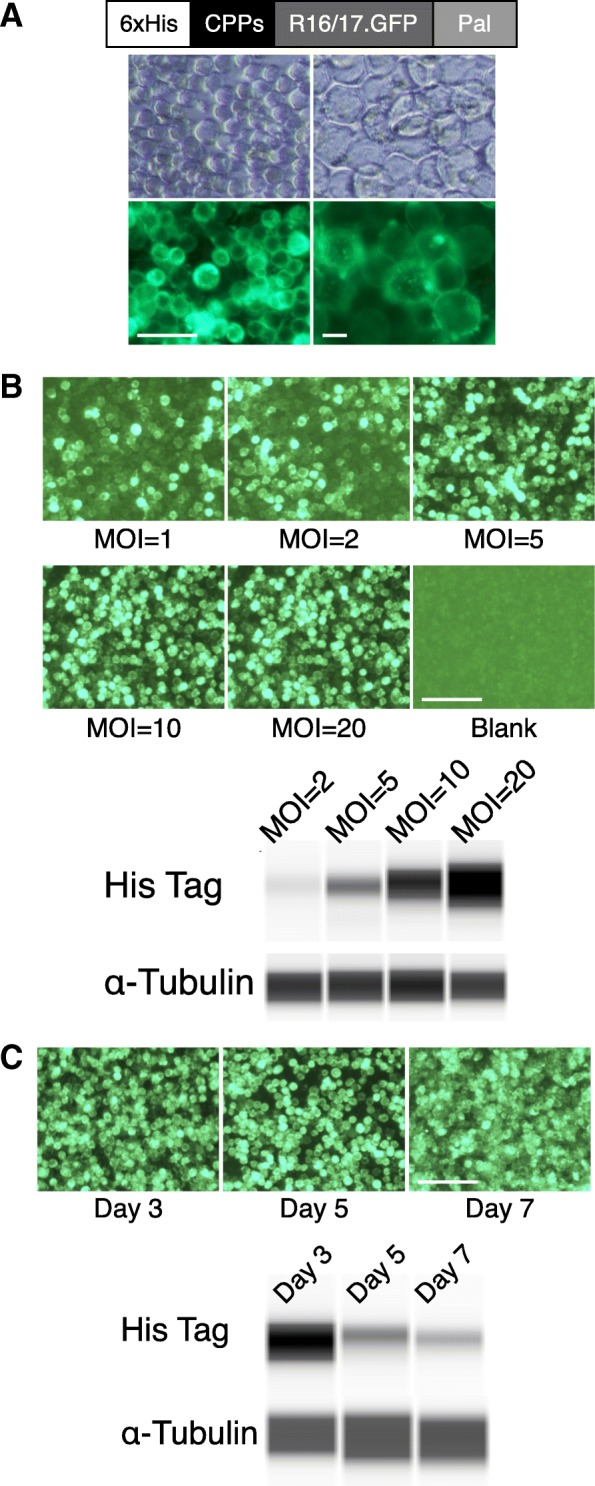
Dystrophin R16/17.GFP.Pal protein is expressed in insect cells and localized at the cell membrane. **a** Configuration of recombinant dystrophin R16/17.GFP.Pal protein and R16/17 protein expression in insect cells. Top panel: light microscopy; Bottom panel: fluorescence microscopy for GFP signal; Left panel: 10X; Right panel: 20X. The dystrophin R16/17.GFP.Pal protein was successfully expressed in the baculovirus-insect cell system and localized at the cell membrane. Left scale bar = 50 μm; Right scale bar = 10 μm; **b** Optimal MOI was determined for R16/17 protein production. The GFP signal was observed on day one after baculoviruses infection of *High Five* insect cells with different MOIs. The expression level from different MOIs was examined by western blot with an anti-His tag antibody, and α-tubulin serves as the loading control. Scale bar = 50 μm; **c** Optimal harvesting time was determined for R16/17 protein production. *High Five* cells were infected with baculoviruses (MOI = 20). The GFP signal and the expression of R16/17.GFP protein were examined 3, 5 and 7 days after virus infection by fluorescence microscopy and western blot with an anti-His tag antibody, respectively. The level of α-tubulin is used for the loading control. The highest protein expression was found 3 days after virus infection. Scale bar = 50 μm. Western blot was performed with ProteinSimple WES capillary western blot system

We exploited baculovirus-insect cell protein expression system to express recombinant dystrophin R16/17 protein. To generate baculoviral plasmids encoding R16/17.GFP.Pal protein (Fig. 1a), we chose the pFASTBAC1 plasmid (Invitrogen, Grand Island, NY, USA) as the backbone plasmid. The fragment of R16/17.GFP.Pal was amplified from the plasmid AAV.R16/17.GFP.Pal (YL299) we reported previously (Lai et al. 2013) using the primers 5′-gcgcactagtcgccaccATG*catcaccatcaccatcac*gaaatttcttatgtgccttctacttatttgactg-3′ (underlined: *Spe I*; uppercase: start codon; italic: 6xHis tag) and 5′-gcgcctcgagttacataatcacgcatttggttttgctcttc-3′ (underlined: *Xho I*), and was subcloned into pFASTBAC1. The resultant plasmid (YL393) carries 6xHis.R16/17.GFP.Pal and serves as an intermediate plasmid for further engineering. Five common CPPs (Additional file 1: Figure S1), including TAT protein transduction domain, a TAT mutant (mTAT), deca-arginine (R10), the CPPs from antennapedia homeodomain (ANTP) and flock house virus coat protein (FHV), were individually inserted between His tag and R16 of the YL393 plasmid to generate the respective CPP-R16/17.GFP.Pal construct. To increase the flexibility of the CPPs, the CPPs are flanked by 4 glycine residues. The sequence of all the plasmids was validated by sequencing.

Baculoviral plasmids were transformed into DH10Bac competent cells (Invitrogen, Grand Island, NY, USA), which contain the bacmid and a helper plasmid. DH10Bac competent cells grow on LB agar plates overlain with X-gal solution and supplemented with 50 μg/ml kanamycin, 7 μg/ml gentamicin, 10 μg/ml tetracycline, and 40 μg/ml IPTG. Single white colonies were inoculated into LB broth with 50 μg/ml kanamycin, 7 μg/ml gentamicin, and 10 μg/ml tetracycline. The recombinant bacmid DNA was isolated from cell culture by using Purelink HiPure plasmid DNA Miniprep kit (Invitrogen, Grand Island, NY, USA). PCR was carried out to confirm that the transgene was incorporated into the recombinant bacmid by using the primers 5′-cccagtcacgacgttgtaaaacg-3′ and 5′-agcggataacaatttcacacagg-3′.

### Cell culture

An insect cell line *sf9* was cultured in SF-900 II serum-free medium (Invitrogen, Grand Island, NY, USA) supplemented with 2% fetal bovine serum (FBS) and 10 μg/ml gentamicin, and incubated in a non-humidified incubator at 28 °C. The recombinant bacmid DNA was transfected to *sf9* cells in a six-well plate by Cellfectin II (Invitrogen, Grand Island, NY, USA) according to the manufacture manual. The supernatant was harvested 7 days after transfection as P1 viral stock. The baculovirus titer was quantified by real-time PCR using the primers: 5′-aagcagcacgacttcttcaagtc-3′ and 5′-ggctgttgtagttgtactccag-3′. To amplify viral stock, P1 viral stock was used to infect *sf9* cells in T175 flasks. The supernatant was harvested 72 h after infection as P2 viral stock and the viral titer was determined by the aforementioned method.

### Recombinant protein production and purification

Another insect cell line *High Five* was used to produce large amount of recombinant proteins. *High Five* cells were cultured in Express Five serum-free medium (Invitrogen, Grand Island, NY, USA) supplemented with 5% FBS, 10 μg/ml gentamicin and 18 mM L-glutamine, and incubated in a non-humidified incubator at 28 °C. Optimal conditions for protein production were empirically established. Capillary western blot was used to detect recombinant R16/17 protein. Briefly, cell lysates with different MOIs were harvested at different time points. The expression of recombinant R16/17 protein was detected with a mouse anti-6xHis tag antibody (1:10, ab18184, Abcam) by ProteinSimple WES capillary western blot system according to a previously published method (Beekman et al. 2014). α-tubulin (mouse anti-α-tubulin antibody, 1:20, T5168, Sigma-Aldrich) was used as the loading control.

To scale up protein production, P2 viral stock was added to *High Five* cells in T175 flasks at a multiplicity of infection (MOI) of 20. Cells were harvested 3 days after infection, and the recombinant proteins were purified from cell lysates with the Probond purification system (Invitrogen, Grand Island, NY, USA). The identities of recombinant proteins were confirmed by western blot using anti-R17 and anti-His tag antibodies.

### Animal care and experiments

Animal experiments were approved by the Animal Care and Use Committee of the University of Missouri. All the animal procedures were in accordance with NIH guidelines. Three strains of mice were used in the study including normal BL6 mice, dystrophin-null *mdx4cv* mice and ∆R4 transgenic mice. Both *mdx4cv* and ∆R4 transgenic mice were on the BL6 background. BL6 and *mdx4cv* mice were originally purchased from the Jackson Laboratory. The experimental mice were generated in house in a barrier facility. Only male mice were used in the study. Age-matched mice were used in all the studies. Generation of ΔR4 micro-dystrophin transgenic mice has been reported before (Crawford et al. 2000; Harper et al. 2002).

For the experiment of comparing transduction efficiency of five CPPs, the recombinant proteins were administered intraperitoneally (IP) to 6 to 8-week-old male ΔR4 mice at the dose of 10 μg/g body weight for six consecutive days (*n* = 4 animals for each group) (Additional file 2: Figure S2A) (Schwarze et al. 1999; Sonnemann et al. 2009). On day seven, protein delivery was given by tail vein injection. TA muscles were harvested one day after tail vein injection.

For muscle function and blood perfusion experiment, mTAT.R16/17.GFP.Pal protein was administered to ΔR4 mice by IP injection daily for 6 days at the dose of 10 μg/g body weight. Then injections were performed twice per week for 6 weeks (*mdx4cv*, *n* = 10; untreated ∆R4, *n* = 38; R16/17 protein treated ∆R4, *n* = 29). The last injection was given by tail vein injection. After the last injection, injected ΔR4 mice were subjected to treadmill challenge and muscle force measurements (untreated ∆R4 without treadmill, *n* = 12; untreated ∆R4 with treadmill, *n* = 10; R16/17 protein treated ∆R4 without treadmill, *n* = 6; R16/17 protein treated ∆R4 with treadmill, *n* = 8) or blood perfusion experiments (*mdx4cv*, *n* = 10; untreated ∆R4, *n* = 16; R16/17 protein treated ∆R4, *n* = 15) (Additional file 2: Figure S2B and S2C). Tissues were harvested after functional assays.

### Immunofluorescence and immunohistochemical staining

All the muscle samples were embedded in Tissue-Tek OCT (Sakura Finetek) immediately after harvesting, and snap-frozen in 2-methylbutane with liquid nitrogen. Histology studies were performed on eight μm cryo-sections. The distribution of R16/17.GFP.Pal protein in the muscle was revealed by detecting GFP and R17. GFP was detected by direct visualization under fluorescence microscopy and immunofluorescence staining with a rabbit-anti-GFP antibody (1:50, a gift from Dr. Yihong Ye, National Institute of Diabetes and Digestive and Kidney Diseases, USA). The R17 domain was examined by immunostaining with a mouse anti-R17 antibody (1:500, a gift from Dr. Glenn Morris, The Robert Jones and Agnes Hunt Orthopedic Hospital, Oswestry, Shropshire, United Kingdom). Sarcolemmal nNOS was identified by immunofluorescence staining with a rabbit anti-C-terminus of nNOS antibody (1:8000, N7280, Sigma-Aldrich), and nNOS activity staining as we published before (Lai et al. 2014). For nNOS activity staining, 16-μm cryosections were cut and fixed in 4% paraformaldehyde for 2 h at 4 °C. Then the tissue sections were permeabilized with 0.2% Triton X-100 at 37 °C for 20 min. The nicotinamide adenine dinucleotide phosphate (NADPH) diaphorase activity of nNOS was revealed by adding the mixture of 0.2% Triton X-100, 0.2 mM NADPH, and 0.16 mg/ml nitroblue tetrazolium (N6876-100MG, Sigma-Aldrich). nNOS activity appears blue staining under the bright field (Hope et al. 1991; Lai et al. 2009, 2013). Dystrophin-associated glycoprotein complex (DGC) components in the muscle of ΔR4 mice were inspected with the following immunofluorescence staining protocol: ΔR4 micro-dystrophin with a mouse anti-H1 antibody (1:20, NCL-DYS3, Novocastra); the full-length mouse dystrophin in the BL6 muscle with a mouse anti-R17 antibody (1:20, a gift from Dr. Glenn Morris, The Robert Jones and Agnes Hunt Orthopedic Hospital, Oswestry, Shropshire, United Kingdom); α-syntrophin with a rabbit anti-syntrophin (1:100, a gift from Dr. Stanley Froehner, University of Washington). For immunofluorescence staining, omission of the primary antibody was used as the control. Alexa Fluor 594-conjugated Goat anti-Mouse (1:100, A-11020, Thermo Fisher Scientific) or Alexa Fluor 594-conjugated Goat anti-Rabbit secondary antibody (1:100, A-11072, Thermo Fisher Scientific) was used for immunofluorescence staining. CD4+ and CD8+ T cell were detected by immunohistochemical staining using our published protocols (Lai et al. 2014). Biotin conjugated Goat anti-Rat secondary antibody (1:1000, R40015, Caltag Laboratories) was added for immunohistochemical staining.

Intensity of sarcolemmal immunolabelling was quantified using a previously published method (Arechavala-Gomeza et al. 2010). Briefly, the intensity of a region containing a portion of the cytoplasm and of the sarcolemma was measured with Image J software. Then, for each of the antibodies, the intensity of staining was obtained by subtracting the minimum intensity value, which represents the cytoplasm or background intensity, from the maximum intensity value, which corresponds to the sarcolemma. For each group, at least 40–50 myofibers from eight muscle sections of two mice were quantified. Data are presented in scatter plots.

### Treadmill exercise and contractile properties of the TA muscle

When sarcolemmal nNOS is lost, long-term treadmill exercise leads to muscle force reduction (Li et al. 2010; Zhang et al. 2013). In this study, treadmill exercise protocol was implemented as follows: Mice run on a horizontal treadmill for 8 weeks. Before starting treadmill exercise, mice are given the treadmill training to make the mice comfortable with the exercise speed. At the start of the treadmill exercise, mice run at 8 m/min for 10 min and then run at 12 m/min for 30 min. The mice run two sessions a week for a total of 8 weeks. Immediately after the eight-week treadmill exercise, specific twitch force and specific tetanic force of the TA muscle were examined using our published method (Li et al. 2011). Another group of mice (age and gender-matched) that did not have treadmill exercise was used as controls for comparison. TA muscle function from exercised mice was compared to non-exercised mice to determine whether treadmill exercise impaired the muscle function.

### Blood flow of the TA muscle

To examine the therapeutic benefits of sarcolemmal nNOS restoration, we measured the blood perfusion of the TA muscle according to our published protocol (Lai et al. 2009). Briefly, *mdx4cv*, non-injected ΔR4 mice and ΔR4 mice injected with R16/17 protein were anesthetized by intraperitoneally injecting the anesthetic mixture of Ketamine (25 mg/ml) and Xylazine (2.5 mg/ml). Body temperature was kept constant at around 37 °C by putting the operating mice on a thermostatically controlled heating pad. Mean arterial pressure (MAP) was monitored by a catheter in the left carotid artery. Hind limb blood flow (HBF) was recorded via a transit-time ultrasound flow probe (0.5 V, 0.7 mm, Transonic Systems, Ithaca, NY 14850 U.S.A.), placed around the femoral artery between the inguinal ligament and the first major branch point. Then, both proximal and distal tendon of left TA muscle and left sciatic nerve were dissected. The distal tendon of the left TA muscle was connected to a force transducer (Aurora Scientific Inc). The contractile properties of left TA muscle were acquired by stimulating left sciatic nerve using a model S48 Grass stimulator (Natus Neurology Incorporated, Middleton, WI 53562 U.S.A.), and the Powerlab data acquisition system (ADInstruments, Castle Hill, Australia).

Hemodynamic parameters were acquired with the Powerlab system. First, MAP and right HBF were recorded as the resting hemodynamics. The left TA muscle was stimulated to contract, and the muscle force was measured according to a published protocol (Li et al. 2011). Lastly, the left TA muscle was subject to a tetanic contraction protocol for 5 min by applying 200 ms tetanic stimulation at the frequency of 100 HZ and using 5 V and 2 ms pulse duration and 15 tetani per min (TPM).

At the end of the tetanic contraction, 0.1 ml of stable-isotope labeled microspheres (15 μm, C-15H10, Gold *STERI*spheres, BioPAL, Worcester, MA 01603 U.S.A.) were injected through the catheter in the left carotid artery. Then MAP and left HBF were measured as the contracting hemodynamics. Both TA muscles, the remaining hind limbs and both kidneys were harvested and weighed. The radioactivity of these tissues was assayed by neutron activation service offered by BioPAL and documented as Disintegrations per minute (*dpm*).

The radioactivity of individual tissues was normalized to their weight. The ratios of radioactivity of the left to right kidney were determined to reflect the even distribution of microspheres in the systemic circulation. TA blood flow was calculated as the following equation: femoral artery blood flow*(normalized TA radioactivity)/(normalized TA radioactivity+normalized hindlimb radioactivity). Right TA blood flow was used as the resting hemodynamics, while blood flow of left TA muscle was used as the contracting hemodynamics.

### Statistical analysis

The data were analyzed with the software GraphPad Prism 6.0a for Mac OS X (GraphPad Software, La Jolla, CA, USA). Comparison between two groups was performed with student t test. Multiple groups comparison was done by analysis of variance (ANOVA). Tukey’s test was used as the post-hoc test to compare the difference between two groups. The statistical significance was considered when the *P* value is less than 0.05.

GraphPad Prism 6.0a, which uses analysis of covariance (ANCOVA) to compare the slopes of the linear regression lines, was used to analyze the data from the blood flow test. It has been revealed that the magnitude of the hemodynamics change is related to the initial resting values (Millar et al. 2007; Baross et al. 2012). Hence, ANCOVA was implemented to determine whether there was a significant difference in the contracting TA blood flow of three groups compared to the resting measures, using the resting values as the covariate. Then post-hoc analysis (Tukey’s test) was used to further determine significant difference between two groups. Linearity of data was confirmed by passing residual normality test (Kolmogorov-Smirnov test) and random distribution of residuals in residual plots (Additional file 3: Figure S3). The statistical significance was considered when the *P* value is less than 0.05.

## Results

### Design and production of recombinant R16/17 protein

To produce recombinant dystrophin R16/17 protein, we engineered the following components in the expression construct including the polyhistidine tag (6xHis) at the N-terminus to help protein purification with nickel (Ni) affinity chromatography, a cell-penetrating peptide (CPP) between the 6xHis tag and the R16 domain to convey the protein across the muscle cell membrane, the GFP tag fused in-frame to the C-terminus of R17 domain for easy tracing of the recombinant protein, and a palmitoylation motif (Pal) at the C-terminus of GFP to target the R16/17 protein to the cell membrane since only membrane-localized R16/17 can anchor nNOS to the sarcolemma (Fig. 1a) (Lai et al. 2013).

Palmitoylation is a post-translational modification unique to eukaryotic cells. For this reason, we used the baculovirus-insect cell system to produce recombinant R16/17.GFP.Pal protein. As shown in Fig. 1a, R16/17.GFP.Pal protein was successfully expressed in the *sf9* insect cells. Importantly, it was localized at the cell membrane as expected. To produce a large quantity of recombinant R16/17 protein for protein therapy, we switched to another insect cell line called *High Five* cells. We found the multiplicity of infection (MOI) of 20 and the harvesting time at 3 days post-transfection resulted in the highest protein yield (Fig. 1b and c). These optimized parameters were used for large-scale protein production.

### mTAT CPP was the most efficient CPP in delivering R16/17.GFP.Pal protein to the muscle of ΔR4 mice

**Fig. 2:**
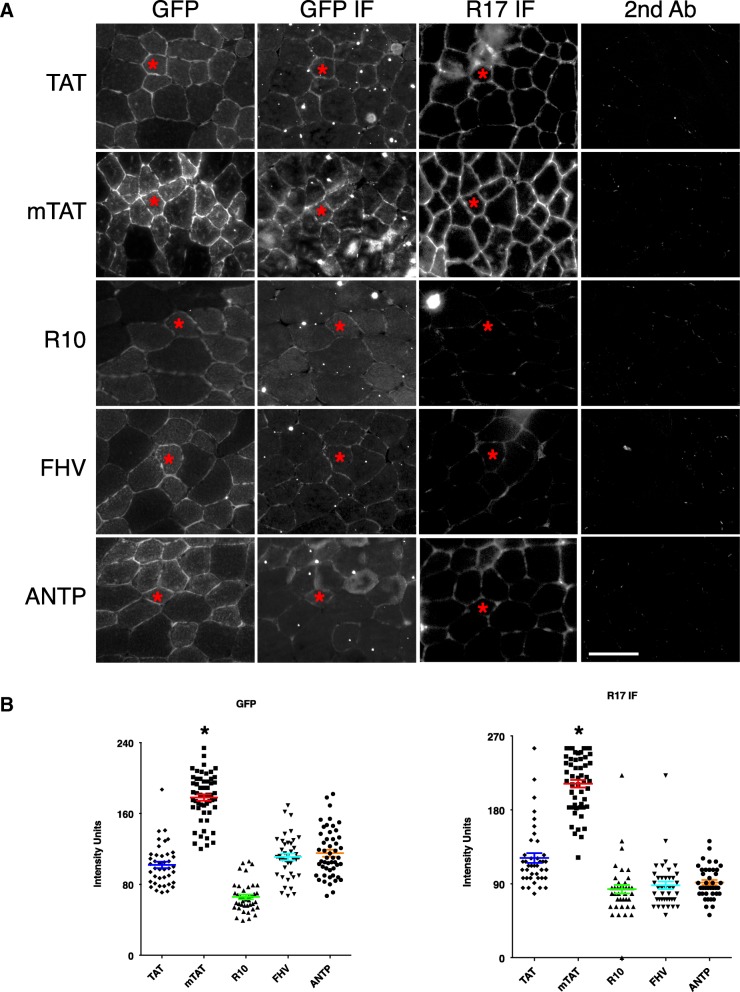
mTAT is the most efficient CPP in transferring R16/17.GFP.Pal protein to the muscle of ΔR4 mice. **a** Transduction efficiency of five different CPPs, including TAT, mTAT, R10, FHV and ANTP, in delivering R16/17.GFP.Pal protein to the muscle of ΔR4 mice was compared. R16/17.GFP.Pal protein carrying different CPPs was injected to ΔR4 mice, and the distribution of R16/17.GFP.Pal protein in the TA muscle was evaluated by direct fluorescence microscopy for GFP signal, and immunofluorescence staining for GFP and R17 on serial sections of the TA muscle. As the images shown here, mTAT is the most efficient CPP in transferring R16/17.GFP.Pal protein to TA muscles of ΔR4 mice. Asterisk: the same myofiber on serial sections. Scale bar = 50 μm; **b** The intensity of the signal of GFP and R17 immunostaining was quantified and confirmed that mTAT led to the highest distribution of R16/17 protein in the muscle

To determine which CPP yielded the best transduction in muscle, we produced R16/17.GFP.Pal protein with five different CPPs, including trans-acting activator of transcription (TAT) protein transduction domain, a TAT mutant (mTAT), deca-arginine (R10), the CPPs from antennapedia homeodomain (ANTP) and flock house virus coat protein (FHV) (Additional file 1: Figure S1). Recombinant proteins were systematically administered to ΔR4 mice. These mice expressed ΔR4-R23/ΔC micro-dystrophin in their skeletal muscle. Since R16/17 is deleted in ΔR4-R23/ΔC micro-dystrophin, nNOS is not localized to the sarcolemma in these mice. A previous study suggested that intraperitoneal injection (IP) of TAT.Lac Z protein can efficiently transfer the Lac Z protein to the muscle (Schwarze et al. 1999). Interestingly, CPP-mediated protein transfer demonstrates a rapid clearance from the blood (Sarko et al. 2010). Hence, we adapted the administration regimen by implementing six daily IP injections followed by an intravenous injection (IV) on day seven at the dosage of 10 μg/g body weight (Additional file 2: Figure S2A). One day after IV injection, muscles were harvested and R16/17.GFP.Pal expression was evaluated by direct visualization of the GFP signal under a fluorescence microscope, and by immunostaining against GFP and R17 (Fig. 2a). To compare transduction efficiency, we took all the images with the same conditions (exposure, gain and offset), and quantified the signal intensity. Although all five CPPs could transfer R16/17.GFP.Pal protein to muscle, mTAT yielded the highest transduction efficiency (Fig. 2b).

### Systemic delivery of the mTAT.R16/17.GFP.Pal protein resulted in body-wide skeletal muscle transduction in ΔR4 mice

**Fig. 3:**
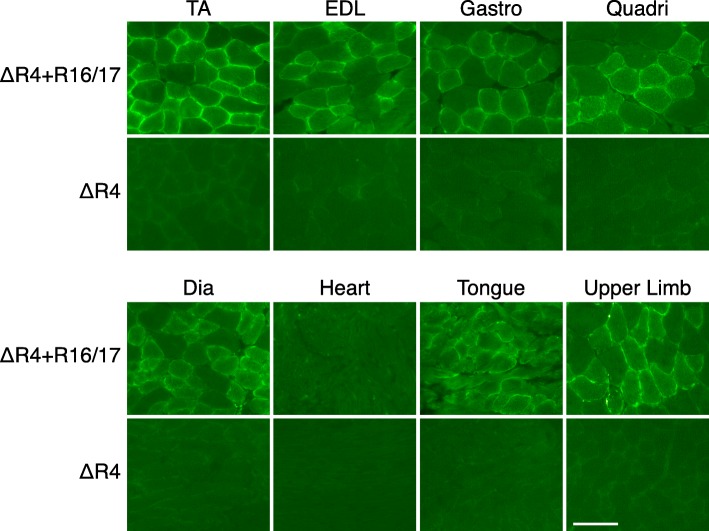
Body-wide distribution of mTAT.R16/17.GFP.Pal protein in ΔR4 mouse muscles. The mTAT.R16/17.GFP.Pal protein was systemically delivered to ΔR4 mice by IP and IV injections. The distribution of mTAT.R16/17.GFP.Pal protein in muscles was evaluated by GFP signal. As shown here, the GFP signal in skeletal muscles, including the TA, EDL, Gastro, Quadri, Dia, Tongue and upper limb muscles, was higher than in the heart. TA: tibialis anterior; EDL: extensor digitorum longus; Gastro: gastrocnemius; Quadri: quadriceps; Dia: diaphragm. Scale bar = 50 μm

Previously, six twice-weekly IP injections of TAT.micro-utrophin protein into *mdx* mice were shown to successfully deliver the micro-utrophin protein to the dystrophic muscle (Sonnemann et al. 2009). To determine therapeutic benefit of the mTAT.R16/17.GFP.Pal protein, we delivered the recombinant protein by IP injection for a total of 7 weeks at the dosage of 10 μg/g body weight, daily injection in the first week and then two injections per week for the remaining 6 weeks (Additional file 2: Figure S2B and S2C). To study body-wide muscle transduction efficiency, we harvested the tibialis anterior (TA), extensor digitorum longus (EDL), gastrocnemius (Gastro), quadriceps (Quadri), diaphragm (Dia), tongue, upper limb muscles and the heart and evaluated the intensity of the GFP signal (Fig. 3). The GFP signal was readily detected in the diaphragm, tongue, and all limb muscles but barely in the heart (Fig. 3). Despite body-wide detection of R16/17 protein in skeletal muscle, we did not see infiltration of CD4+ and CD8+ T cells on immunohistochemical staining (Additional file 4: Figure S4).

### Sarcolemmal nNOS is restored by mTAT.R16/17.GFP.Pal protein transfer in ΔR4 mice

**Fig. 4:**
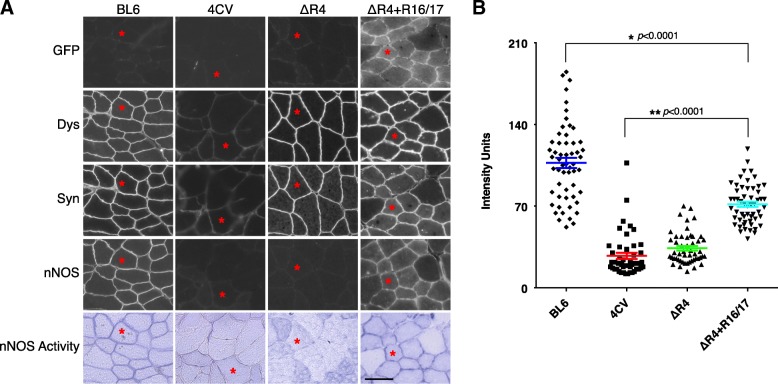
Sarcolemmal nNOS is successfully restored by systemic delivery of mTAT.R16/17.GFP.Pal protein in ΔR4 mice. **a** After systemic delivery of mTAT.R16/17.GFP.Pal protein in ΔR4 mice, dystrophin (Dys), syntrophin (Syn) and nNOS were examined on serial sections of the TA muscle. The GFP signal confirmed that mTAT.R16/17.GFP.Pal was transferred to the TA muscle. With R16/17 protein transfer, sarcolemmal nNOS was successfully restored, as shown by nNOS immunostaining and nNOS activity staining at the muscle membrane. Without R16/17 injection, despite the presence of ΔR4 micro-dystrophin and syntrophin at the sarcolemma, sarcolemmal nNOS was still absent. Asterisk: the same myofiber on serial sections. Scale bar = 50 μm. **b** The signal intensity of nNOS immunostaining was quantified and analyzed by ANOVA. After R16/17 protein transfer, compared to *mdx4cv* and non-injected ΔR4 mice, sarcolemmal nNOS in ΔR4 mice was statistically improved (** *p* < 0.0001). Sarcolemmal nNOS in the muscle of BL6 is significantly higher than *mdx4cv*, ΔR4 and ΔR4 + R16/17 (* *p* < 0.0001)

The main goal of this study is to restore sarcolemmal nNOS in the muscle of ΔR4 mice by R16/17 protein therapy. We examined muscle serial sections for GFP, dystrophin (using a hinge 1 specific antibody), syntrophin, nNOS and nNOS activity in the TA muscle. In line with our previous study (Yue et al. 2006), ΔR4 micro-dystrophin and α1-syntrophin were localized at the muscle membrane. Sarcolemmal nNOS was absent in untreated ∆R4 mice. On the contrary, in ΔR4 mice treated with R16/17.GFP.Pal protein injection, in addition to membrane localization of ΔR4 micro-dystrophin and syntrophin, both immunostaining and nNOS activity staining revealed recovery of nNOS at the sarcolemma (Fig. 4a). Quantification of sarcolemma nNOS suggests that there is no significant difference of the sarcolemmal nNOS level between *mdx4cv* and non-injected ΔR4 mice. After R16/17.GFP.Pal protein delivery, sarcolemmal nNOS level was more than doubled in ΔR4 mice (*p* < 0.0001) (Fig. 4b).

### Recovering sarcolemmal nNOS by R16/17 protein therapy prevented force reduction induced by long-term treadmill running in ΔR4 mice, and improved muscle perfusion during exercise

**Fig. 5:**
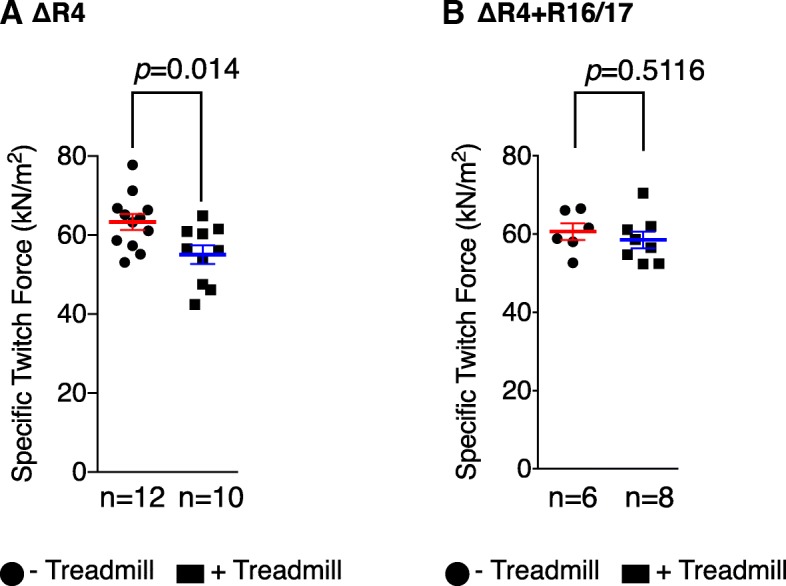
R16/17.GFP.Pal protein transfer improves muscle function of ΔR4 mice. ΔR4 mice injected with R16/17 protein were subjected to long-term treadmill exercise. Immediately following treadmill exercise, in situ TA muscle function assay was performed to measure contractile properties of the TA muscles and compared to non-exercised mice. Without R16/17 protein transfer, treadmill exercise caused significant reduction of specific twitch force (**a**), but with R16/17 protein transfer, specific twitch force was maintained (**b**)

To further study the therapeutic benefit of R16/17 protein-mediated sarcolemmal nNOS recovery, we challenged ∆R4 mice that either received or did not receive R16/17 protein therapy with continuous treadmill exercise for a total of 8 weeks, and then compared the specific twitch force and the specific tetanic force of the TA muscle between the mice that were and were not challenged with treadmill running. Eight weeks after the stop of protein injection, we still observed persistent presence of the GFP signal at the sarcolemma in R16/17 protein injected ∆R4 mice. Immunofluorescence staining with R16 and R17-specific antibodies further confirmed the presence of R16/17 protein at the sarcolemma. Importantly, sarcolemmal nNOS recovery was confirmed by immunofluorescence staining (Additional file 5: Figure S5). Either for non-injected or for R16/17 protein-injected ΔR4 mice, there is no significant change in the specific tetanic force between exercised and non-exercised muscle (Additional file 6: Figure S6). However, for ∆R4 mice that did not receive R16/17 protein therapy, their specific twitch force was 63.33 ± 2 kN/M^2^ in the absence of continuous treadmill challenge. While challenged with treadmill running, the specific twitch force dropped to 55.03 ± 2.36 kN/M^2^ (*p* = 0.014) (Fig. 5a). This force drop was not detected in ∆R4 mice that received R16/17 protein therapy (*p* = 0.5116) (Fig. 5b). This result is consistent with the previous studies that reduction of muscle force after chronical treadmill exercise is observed in the mice without sarcolemmal nNOS but not in the mice with sarcolemmal nNOS such as wild-type mice or nNOS-restoring mini-dystrophin transgenic mice (Li et al. 2010; Zhang et al. 2013).

**Fig. 6:**
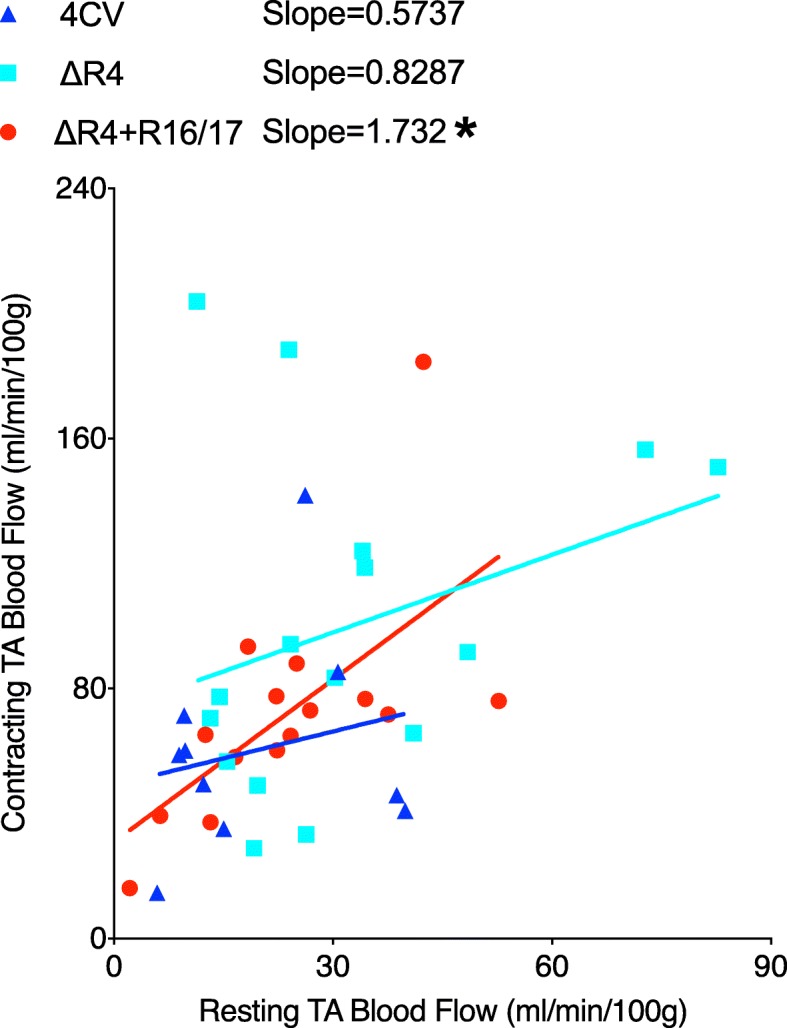
R16/17 protein transfer improves blood perfusion of the contracting muscle. Blood flow of the TA muscles was measured by microsphere deposition and the flow probe reading. The linear regression model was established by plotting the values from the contracting muscle against those from the resting muscle. Statistical analysis was performed to compare the slopes of three experimental groups: *mdx4cv mice*, ΔR4 mice without R16/17 protein transfer and ΔR4 mice with R16/17 protein transfer. *: ΔR4 mice treated with R16/17 protein showed a slope that was significantly higher than the other two groups, indicating improved perfusion in the contracting TA muscle in this group of mice

During muscle contraction, NO synthesized by sarcolemmal nNOS infiltrates into the nearby vasculature and causes vessel dilation to insure sufficient muscle perfusion. To determine whether restoration of sarcolemmal nNOS by the R16/17 protein could improve blood perfusion of the contracting muscle, we measured the blood flow (BF) of the TA muscle by combining microsphere deposition and the flow probe reading. Microsphere deposition in kidneys and TA muscles was determined. Additional file 8: Table S1 shows the animal age, body weight, muscle weight, kidney weight, and left and right kidney microsphere distribution ratio. There was no significant difference in the age and weights among three groups. Microsphere distribution in both left and right kidney was almost same, suggesting of an even distribution of microspheres in the systemic circulation. The blood flow of the contracting and resting TA muscle was obtained by integrating microsphere deposition in the TA muscle and the blood flow of the femoral artery obtained by the flow probe. Regression lines were established by plotting contracting TA BF against resting TA BF. The slope of the lines reflects the rate of increase of TA BF in response to muscle contraction. ANCOVA revealed that the slopes are significantly different among three groups (*p* = 0.0005, ANCOVA), indicating a significant change in the contracting TA BF among three groups. Individual analysis using ANCOVA revealed no significant difference in the slopes for *mdx4cv* and non-injected ΔR4 mice (0.5737 vs 0.8287, *p* = 0.6657). However, there was a significant difference in the slope of ΔR4 mice with R16/17 protein delivery (*mdx4cv* vs ΔR4 + R16/17, 0.5737 vs 1.732, *p* = 0.0013; non-injected ΔR4 vs ΔR4 + R16/17, 0.8287 vs 1.732, *p* = 0.0038). In R16/17 protein treated ∆R4 mice, the slope was significantly increased, suggesting that the R16/17 protein therapy significantly enhanced blood perfusion during muscle contraction (Fig. 6).

## Discussion

In this study, we showed that systemic delivery of a recombinant dystrophin R16/17 protein restored sarcolemmal nNOS in ΔR4 mice, a mouse model for BMD. More importantly, sarcolemmal nNOS restoration by R16/17 protein delivery improved muscle perfusion during contraction and ameliorated functional ischemia-induced muscle damage in ΔR4 mice.

Sarcolemmal nNOS plays a critical role in many aspects of muscle activity and function. In DMD patients and a significant portion of BMD patients, membrane-associated nNOS is lost. Studies from many groups have provided compelling evidence suggesting that the lack of sarcolemmal nNOS contributes to DMD and BMD pathogenesis. For this reason, investigators have explored therapeutic benefits of restoring nNOS in mouse models of DMD by overexpressing nNOS via transgenic approach (Wehling et al. 2001; Wehling-Henricks et al. 2005; Wehling-Henricks and Tidball 2011; Rebolledo et al. 2016), and adeno-associated virus (AAV)-mediated gene therapy (Lai et al. 2014). Collectively, these studies suggest that nNOS over-expression can improve muscle histopathology and muscle function (Wehling et al. 2001; Wehling-Henricks et al. 2005; Wehling-Henricks and Tidball 2011; Lai et al. 2014; Rebolledo et al. 2016). However, direct delivery of nNOS by gene therapy or protein therapy may lead to untoward expression in tissues and cells that normally do not have nNOS, and consequently cause toxicity. An alternative strategy to nNOS over-expression is to deliver nNOS-binding dystrophins to muscle. To this end, we have shown that that R16/17-inclusive truncated dystrophins can successfully restore nNOS to the muscle cell membrane of DMD mice (Lai et al. 2009; Zhang and Dongsheng 2012; Lai et al. 2013; Zhang et al. 2013). Further, dystrophin R16/17-mediated sarcolemmal nNOS recovery prevented functional ischemia, improved muscle force and enhanced exercise performance (Lai et al. 2009; Zhang and Dongsheng 2012; Lai et al. 2013; Zhang et al. 2013).

Previously, we found that AAV-mediated expression of dystrophin R16/17 alone restored sarcolemmal nNOS in ΔH2-R19 mini-dystrophin transgenic mice, in which sarcolemmal nNOS is missing due to the absence of dystrophin R16/17 (Lai et al. 2013). This result suggests that inability of truncated dystrophins to anchor sarcolemmal nNOS can be complemented by delivering dystrophin R16/17 alone. For some BMD or DMD receiving exon skipping or gene editing, due to lack of R16/17, the presence of truncated dystrophins cannot anchor nNOS to the sarcolemma. In this study, we used ΔR4 mice to mimic these patients since this strain of mice expressed an R16/17-deleted micro-dystrophin and did not have sarcolemmal nNOS. Proteins therapy represents an important treatment modality in modern medical practice. Of the 20 top-selling drugs, 9 are proteins (Leader et al. 2008). Here we explored whether delivering dystrophin R16/17 protein can also result in sarcolemmal nNOS localization. Specifically, we tested whether the R16/17 protein therapy can restore sarcolemmal nNOS in ΔR4 mice, a model for BMD.

We first established a baculovirus-insect cell protein production system for highly efficient production of the palmitoylated recombinant R16/17 protein (Fig. 1). We then developed a CPP-mediated approach to deliver the dystrophin R16/17 protein to the muscle of ΔR4 mice. CPPs are short cationic peptides that have been widely used to facilitate the transport of molecules across the cell membranes (Koren and Torchilin 2012). The first successful CPP was called TAT and it was derived from the protein transduction domain of the human immunodeficiency virus TAT protein (Fawell et al. 1994; Schwarze et al. 1999). The Ervasti lab has previously shown that the TAT CPP can mediate efficient muscle transduction of the utrophin proteins for DMD therapy in the dystrophin-null mice (Sonnemann et al. 2009). Others have used the TAT CPP to deliver oligonucleotides for exon skipping therapy for DMD and other muscle diseases (Jearawiriyapaisarn et al. 2008; Wu et al. 2008; Yin et al. 2008; Aoki et al. 2012; Malerba et al. 2012). Several additional CPPs have been developed over the last two decades for various applications. To achieve the best muscle delivery, we screened five most commonly used CPPs including TAT, mTAT, R10, ANTP and FHV (Additional file 1: Figure S1 & Fig. 2). We found mTAT be the most effective CPP in the context of our study. mTAT was generated by rational alignment of arginine residues and substitution of alanine residues to strengthen α-helical backbone of TAT CPP (Additional file 1: Figure S1)(Ho et al. 2001), and it has been shown that mTAT CPP was significantly more efficient than the original TAT CPP in both in vitro and in vivo studies (Ho et al. 2001). Others have shown that efficiency of CPP-mediated protein delivery is cargo-dependent (El-Andaloussi et al. 2007), supporting that mTAT CPP is superior to other CPPs in terms of delivering dystrophin R16/17.GFP.Pal protein (Fig. 2).

With the identification of the mTAT.R16/17.GFP.Pal protein as our best candidate, we tested protein therapy. Muscle is one of the most broadly distributed tissues in the body. An effective therapy for muscle diseases requires body-wide delivery. To this end, we tested whether we can achieve body-wide R16/17 protein delivery in ∆R4 mice. As demonstrated in Fig. 3, intraperitoneal and intravenous injection indeed led to good transduction of most skeletal muscles in the body. Further mTAT.R16/17.GFP.Pal protein did not induce CD4+ and CD8+ T cell infiltration. Interestingly, we did not detect a good protein delivery in the heart. The reason for the poor cardiac delivery is unclear. Future comprehensive studies are needed to fully address the issue of cardiac delivery. It should be noted that since dystrophin does not directly interact with nNOS in cardiomyocytes (Johnson et al. 2012), even if we eventually achieve successful cardiac delivery of the R16/17 protein, it may not restore the homeostasis of nNOS in the heart.

Next, we examined whether our R16/17 protein therapy could restore sarcolemmal nNOS. Consistent with our previous study (Lai et al. 2013), we found excellent correlation between R16/17 protein transduction and sarcolemmal nNOS localization (Fig. 4). Noteworthily, the level of sarcolemmal nNOS recovered by R16/17 protein is significantly lower than that of wild-type muscle (Fig. 4b), suggesting that the amount of R16/17 protein in the muscle is not sufficient for fully restoring sarcolemmal nNOS. Future comprehensive pharmacokinetic studies and optimization of injection regimen are needed to improve the delivery of R16/17 protein to the muscle. Finally, we investigated the potential therapeutic effects of R16/17 protein delivery using two independent assays. We compared the blood flow in contracting and resting muscles of ΔR4 mice that were either treated with R16/17 protein therapy or not treated. We also measured muscle force following continuous treadmill challenging. In both assays, we observed statistically significant benefits of R16/17 protein therapy (Figs. 5 and 6). Specifically, mice that received R16/17 protein therapy showed better muscle perfusion during contraction and they were also effectively prevented from strenuous exercise-induced force loss.

Sarcolemmal nNOS anchoring requires both R16/17 and syntrophin at the muscle cell membrane. In DMD, sarcolemmal syntrophin is lost due to the disassembly of dystrophin-associated protein complex. For this reason, R16/17 protein therapy alone cannot anchor nNOS to the sarcolemma in DMD (Additional file 7: Figure S7B). R16/17 is encoded by exons 42 to 46 of the dystrophin gene. This region happens to locate at the major deletion hot spot in DMD and BMD patients. For DMD patients with deletion in this region, there have been significant efforts to develop exon-skipping therapy and CRISPR gene editing therapy to restore the dystrophin reading frame. However, even if these therapies are successful, they will still not be able to restore nNOS to the sarcolemma. The ∆R4 mice used in this study are reminiscent of BMD patients with in-frame deletion of exons 42–46 or DMD patients with out-of-frame deletion in this region but have been successfully converted to a BMD phenotype by exon-skipping or gene editing (Additional file 7: Figure S7C). The R16/17 protein therapy described here offers a hope to restore the missing sarcolemmal nNOS in these patients (Additional file 7: Figure S7C). Loss of sarcolemmal nNOS is commonly seen in cachexia, aging-related muscular atrophy and several other neuromuscular disorders. Future studies are needed to test our R16/17 protein therapy in animal models of these diseases.

## Conclusions

In conclusion, recombinant dystrophin R16/17 protein can be produced by a baculovirus-insect cell system. The CPP, mTAT, can efficiently transfer recombinant R16/17 protein to the muscle. Delivery of dystrophin R16/17 protein to the muscle of ΔR4 micro-dystrophin transgenic mice successfully restores sarcolemmal nNOS. More importantly, restoration of sarcolemmal nNOS by dystrophin R16/17 protein improves blood perfusion of the contracting muscle and prevents the reduction of muscle force mediated by continuous treadmill exercise. Hence, dystrophin R16/17 protein delivery represents a novel therapeutic approach to recover sarcolemmal nNOS in the muscle with the loss of sarcolemmal nNOS.

## Additional files

Additional file 1: **Figure S1.** The amino acid sequence of five commonly used CPPs. In mTAT, the mutated residues are highlighted in red color. mTAT: TAT mutant. (PDF 220 kb)

Additional file 2: **Figure S2.** Schematic outline of the protein injection and experiment timeline. Black arrow: IP injection; Red arrow: IV injection; Green arrow stands for the experiments reported in Fig. 2; Purple arrow stands for the experiments reported in Fig. 5, and Blue arrow stands for the experiments in Figs. 3, 4 and 6. IP: intraperitoneal; IV: intravenous. (PDF 225 kb)

Additional file 3: **Figure S3.** Residual plots of linear regression lines. Blood flow of the contracting TA muscle from *mdx4cv*, ΔR4 and ΔR4 + R16/17 mice was compared with ANCOVA. First, linear regression models were established by plotting the contracting blood flow against rest blood flow. In the residual plots, residuals from three linear regression lines are randomly scattered along the zero line, supporting linearity of the data from three groups. (PDF 230 kb)

Additional file 4: **Figure S4.** Immunohistochemical staining of CD4 and CD8 T cells in the TA muscle. Apparently, in the muscle of *mdx4cv* mice, there is the infiltration of CD4 and CD8 positive cells. After mTAT.R16/17.GFP protein delivery, no infiltration of CD4, or CD8 positive cells in the muscle was observed. Red arrow: CD4 or CD8 positive cells. (PDF 3664 kb)

Additional file 5: **Figure S5.** The R16/17.GFP protein was detected in the TA muscle at 8 weeks after the stop of protein injection. Dystrophin R16/17.GFP protein in the TA muscle was identified by immunofluorescence staining with the antibodies against GFP, R16 and R17. Sarcolemmal nNOS is also present. The presence of dystrophin R16/17.GFP and sarcolemmal nNOS suggested that R16/17 protein and sarcolemmal nNOS persisted at least 8 weeks after protein delivery. Asterisk: the same myofiber. (PDF 922 kb)

Additional file 6: **Figure S6.** Specific tetanic force of the TA muscle of ΔR4 mice with or without R16/17 protein transfer. For both non-injected (**A**) and R16/17 protein-injected (**B**) ΔR4 mice, there is no significant change of the specific tetanic force between exercised and non-exercised muscle. (PDF 256 kb)

Additional file 7: **Figure S7.** Restoration of sarcolemmal nNOS by dystrophin R16/17 protein transfer in ΔR4 micro-dystrophin mice. Sarcolemmal nNOS anchoring requires both R16/17 and syntrophin at the muscle cell membrane. **A**, Full-length dystrophin anchors nNOS to the muscle cell membrane in the normal muscle; **B**, In DMD, sarcolemmal syntrophin is lost due to disassembly of dystrophin-associated protein complex. R16/17 protein therapy alone cannot restore sarcolemmal nNOS in DMD; **C**, In ΔR4 mice, ΔR4 micro-dystrophin recruits syntrophin to the muscle membrane, and sarcolemmal nNOS is still absent since R16/17 domain is deleted in ΔR4 micro-dystrophin. But with R16/17 protein delivery, sarcolemmal nNOS is recovered in the muscle of ΔR4 mice. (PDF 61 kb)

Additional file 8: **Table S1.** Parameters of blood flow experiment. (DOC 34 kb)

## Data Availability

All other data are available from the corresponding authors upon request.

## Abbreviations

6xHis: polyhistidine tag
ANCOVA: Analysis of covariance
ANOVA: Analysis of variance
ANTP: Antennapedia homeodomain
BF: Blood flow
BMD: Becker muscular dystrophy
CPP: Cell penetrating peptide
Dia: Diaphragm
DMD: Duchenne muscular dystrophy
DPM: Disintegrations per minute
EDL: Extensor digitorum longus
FBS: Fetal bovine serum
FHV: Flock house virus coat protein
Gastro: Gastrocnemius
HBF: Hind limb blood flow
IP: Intraperitoneal injection
IV: Intravenous injection
MAP: Mean arterial pressure
MOI: Multiplicity of infection
mTAT: a TAT mutant
Ni: Nickel
nNOS: Neuronal nitric oxide synthase
NO: Nitric oxide
Pal: A palmitoylation motif
Quadri: Quadriceps
R: Spectrin-like repeat
R10: Deca-arginine
TA: Tibialis anterior
TAT: Trans-acting activator of transcription protein transduction domain
ΔR4: ΔR4-R23/ΔCT
