# Supplementary material for: Dystrophin R16/17 protein therapy restores sarcolemmal nNOS in trans and improves muscle perfusion and function
Source: Mol Med. 2019 Jul 2;25:31. doi: 10.1186/s10020-019-0101-6 (PMC6607532; doi:10.1186/s10020-019-0101-6)
Supplement: Supplementary file 1 — Figure S1. The amino acid sequence of five commonly used CPPs. In mTAT, the mutated residues are highlighted in red color. mTAT: TAT mutant. (PDF 220 kb) [file 10020_2019_101_MOESM1_ESM.pdf]

**TAT**	YGRKKRRQRRR
**mTAT**	YARAAARQARA
**R10**	RRRRRRRRRR
**FHV**	RRRRNRTRRNRRRVR
**ANTP**	RQIKIWFQNRRMKWKK
