# Supplementary material for: Dystrophin R16/17 protein therapy restores sarcolemmal nNOS in trans and improves muscle perfusion and function
Source: Mol Med. 2019 Jul 2;25:31. doi: 10.1186/s10020-019-0101-6 (PMC6607532; doi:10.1186/s10020-019-0101-6)
Supplement: Supplementary file 2 — Figure S2. Schematic outline of the protein injection and experiment timeline. Black arrow: IP injection; Red arrow: IV injection; Green arrow stands for the experiments reported in Fig. 2; Purple arrow stands for the experiments reported in Fig. 5, and Blue arrow stands for the experiments in Figs. 3, 4 and 6. IP: intraperitoneal; IV: intravenous. (PDF 225 kb) [file 10020_2019_101_MOESM2_ESM.pdf]

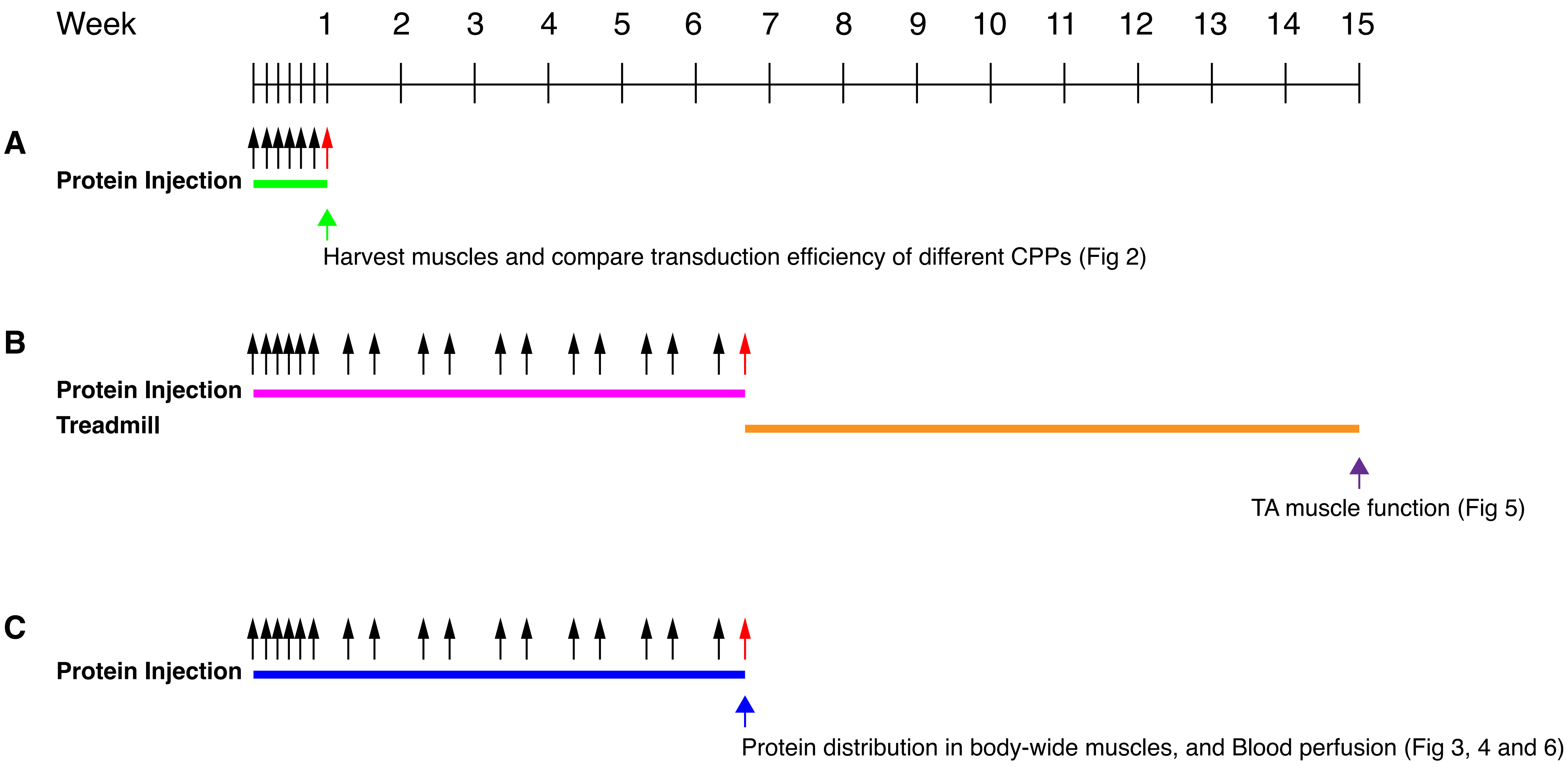
Week
1
2
3
4
5
6
7
8
9
10
11
12
13
14
15
A
Protein Injection
Harvest muscles and compare transduction efficiency of different CPPs (Fig 2)
B
Protein Injection
Treadmill
TA muscle function (Fig 5)
C
Protein Injection
Protein distribution in body-wide muscles, and Blood perfusion (Fig 3, 4 and 6)
