# Supplementary figures and images for: Dystrophin R16/17 protein therapy restores sarcolemmal nNOS in trans and improves muscle perfusion and function
Source: Mol Med. 2019 Jul 2;25:31. doi: 10.1186/s10020-019-0101-6 (PMC6607532; doi:10.1186/s10020-019-0101-6)

**A. 4CV**

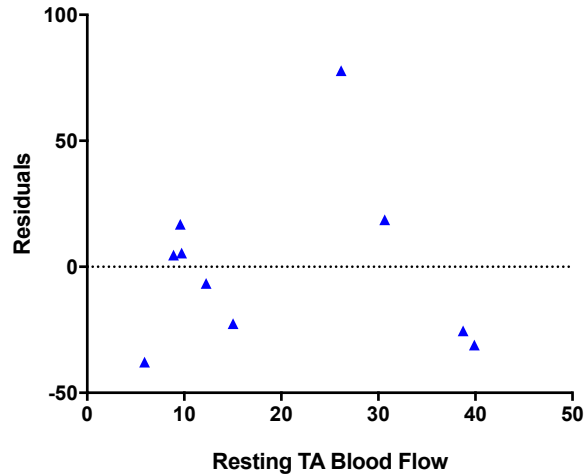

**B.  $\Delta R4$**

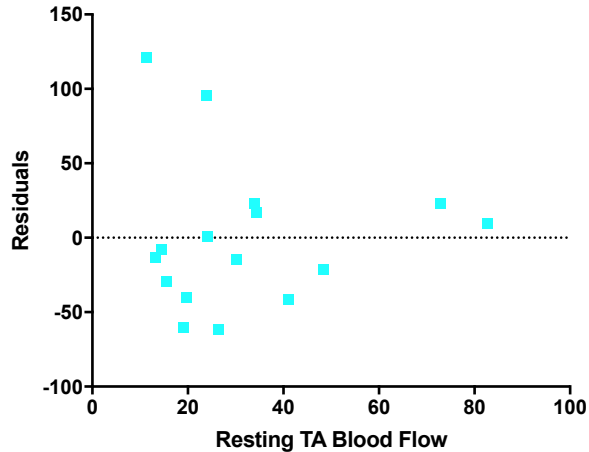

**C.  $\Delta R4 + R16/17$**

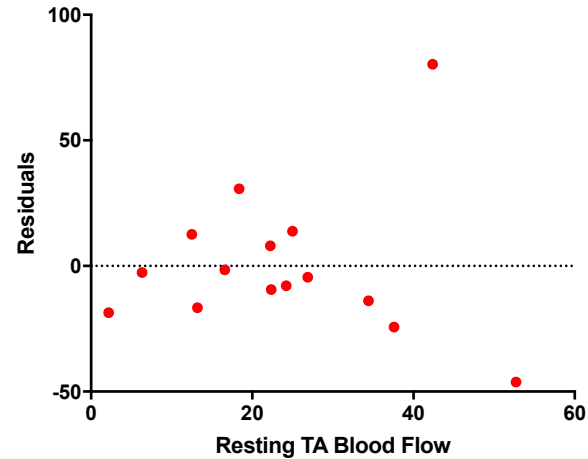

Supplement: Supplementary file 3 — Figure S3. Residual plots of linear regression lines. Blood flow of the contracting TA muscle from mdx4cv, ΔR4 and ΔR4 + R16/17 mice was compared with ANCOVA. First, linear regression models were established by plotting the contracting blood flow against rest blood flow. In the residual plots, residuals from three linear regression lines are randomly scattered along the zero line, supporting linearity of the data from three groups. (PDF 230 kb) [file 10020_2019_101_MOESM3_ESM.pdf]

BL6

4CV

$\Delta R4$

$\Delta R4+mTAT$

CD4

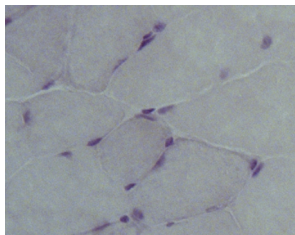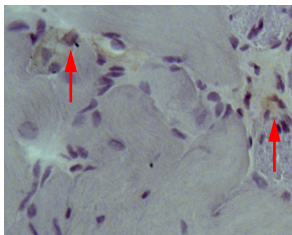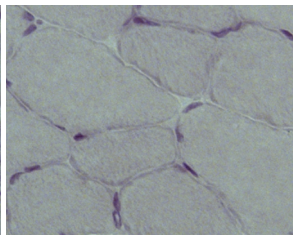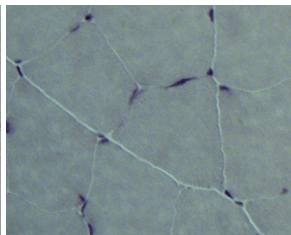

CD8

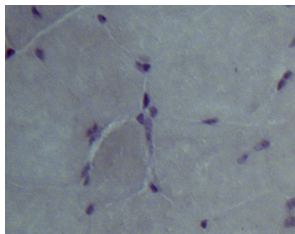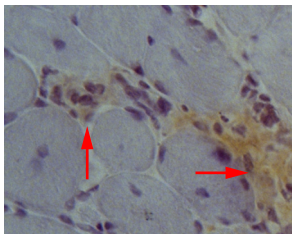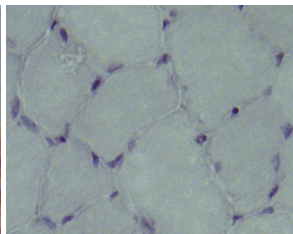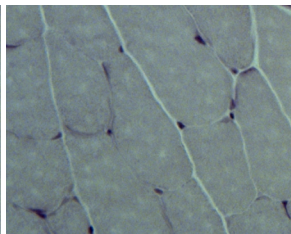

4CV

$\Delta R4+mTAT$

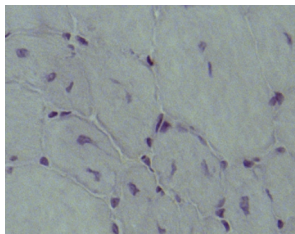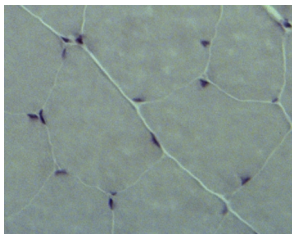

Secondary antibody only

Supplement: Supplementary file 4 — Figure S4. Immunohistochemical staining of CD4 and CD8 T cells in the TA muscle. Apparently, in the muscle of mdx4cv mice, there is the infiltration of CD4 and CD8 positive cells. After mTAT.R16/17.GFP protein delivery, no infiltration of CD4, or CD8 positive cells in the muscle was observed. Red arrow: CD4 or CD8 positive cells. (PDF 3664 kb) [file 10020_2019_101_MOESM4_ESM.pdf]

GFP IF

R16

R17

nNOS

$\Delta R4$

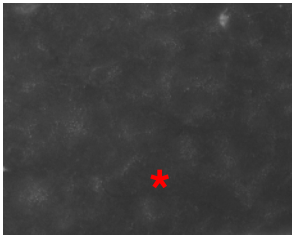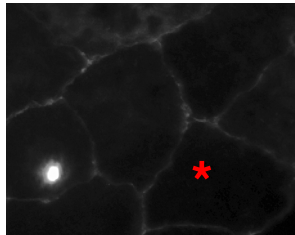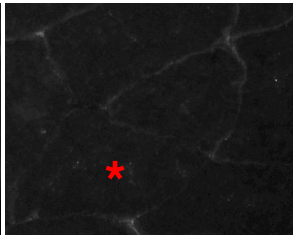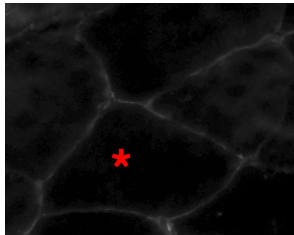

$\Delta R4 + R16/17$

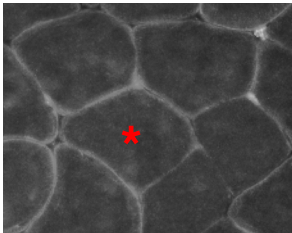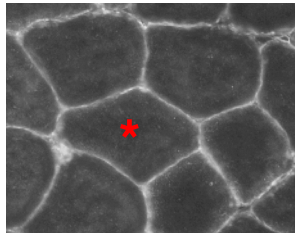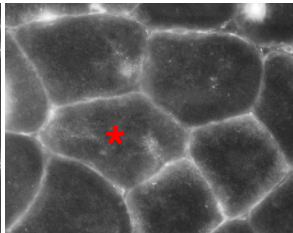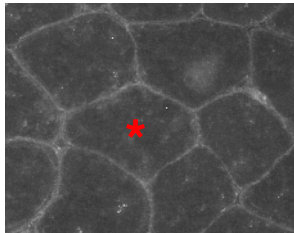

Supplement: Supplementary file 5 — Figure S5. The R16/17.GFP protein was detected in the TA muscle at 8 weeks after the stop of protein injection. Dystrophin R16/17.GFP protein in the TA muscle was identified by immunofluorescence staining with the antibodies against GFP, R16 and R17. Sarcolemmal nNOS is also present. The presence of dystrophin R16/17.GFP and sarcolemmal nNOS suggested that R16/17 protein and sarcolemmal nNOS persisted at least 8 weeks after protein delivery. Asterisk: the same myofiber. (PDF 922 kb) [file 10020_2019_101_MOESM5_ESM.pdf]

## A $\Delta R4$

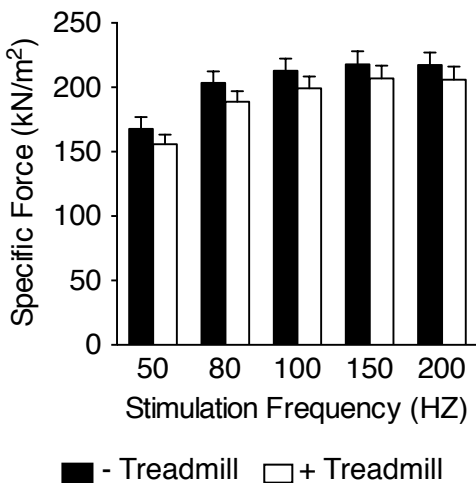

## B $\Delta R4+R16/17$

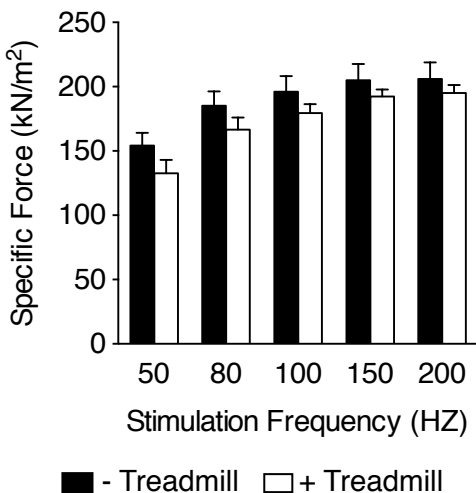

Supplement: Supplementary file 6 — Figure S6. Specific tetanic force of the TA muscle of ΔR4 mice with or without R16/17 protein transfer. For both non-injected (A) and R16/17 protein-injected (B) ΔR4 mice, there is no significant change of the specific tetanic force between exercised and non-exercised muscle. (PDF 256 kb) [file 10020_2019_101_MOESM6_ESM.pdf]

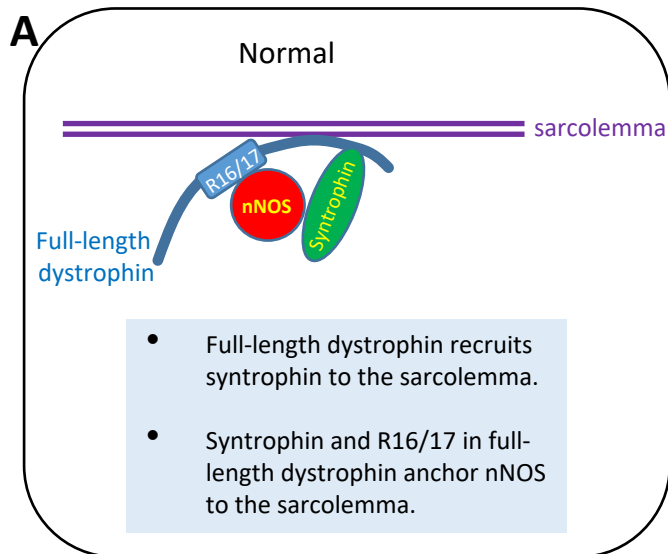

Sarcolemma nNOS anchoring requires **both** R16/17 **and** syntrophin.

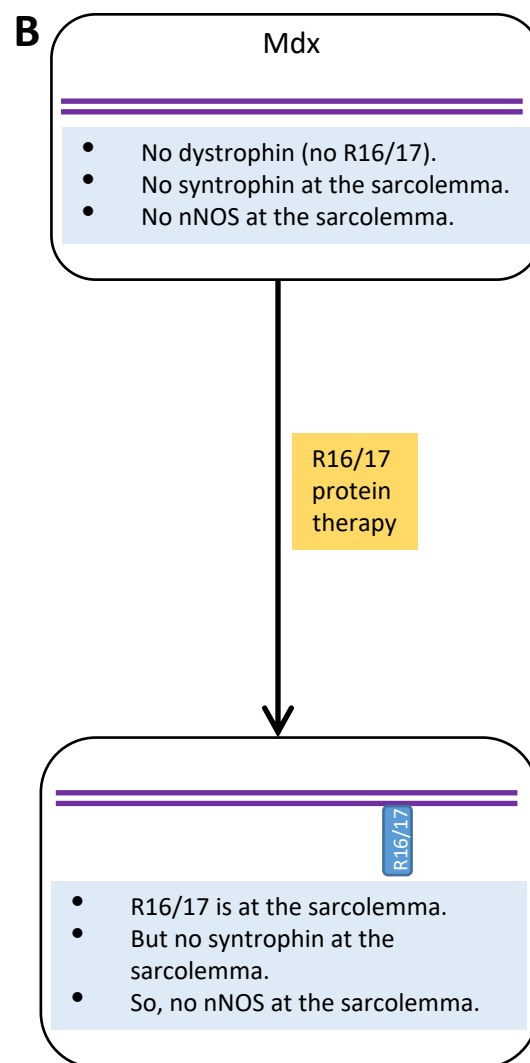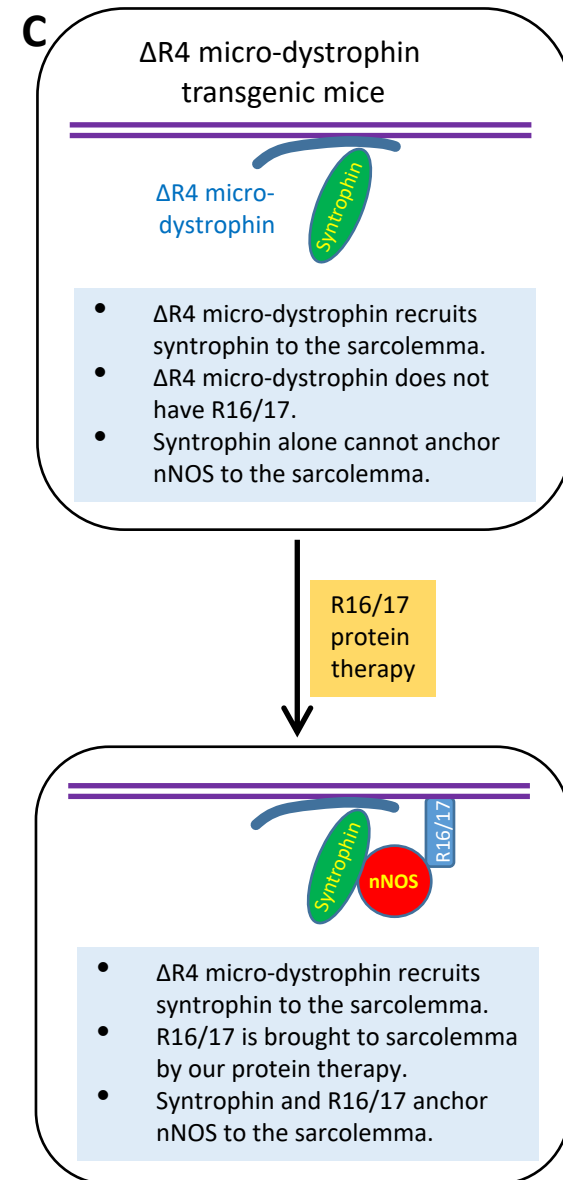

Supplement: Supplementary file 7 — Figure S7. Restoration of sarcolemmal nNOS by dystrophin R16/17 protein transfer in ΔR4 micro-dystrophin mice. Sarcolemmal nNOS anchoring requires both R16/17 and syntrophin at the muscle cell membrane. A, Full-length dystrophin anchors nNOS to the muscle cell membrane in the normal muscle; B, In DMD, sarcolemmal syntrophin is lost due to disassembly of dystrophin-associated protein complex. R16/17 protein therapy alone cannot restore sarcolemmal nNOS in DMD; C, In ΔR4 mice, ΔR4 micro-dystrophin recruits syntrophin to the muscle membrane, and sarcolemmal nNOS is still absent since R16/17 domain is deleted in ΔR4 micro-dystrophin. But with R16/17 protein delivery, sarcolemmal nNOS is recovered in the muscle of ΔR4 mice. (PDF 61 kb) [file 10020_2019_101_MOESM7_ESM.pdf]
