# Supplementary material for: Dystrophin R16/17 protein therapy restores sarcolemmal nNOS in trans and improves muscle perfusion and function
Source: Mol Med. 2019 Jul 2;25:31. doi: 10.1186/s10020-019-0101-6 (PMC6607532; doi:10.1186/s10020-019-0101-6)
Supplement: Supplementary file 8 — Table S1. Parameters of blood flow experiment. (DOC 34 kb) [file 10020_2019_101_MOESM8_ESM.doc]

Table S1. Parameters of blood flow experiment.

|  | *mdx 4cv* (n=10) | ΔR4 (n=16) | ΔR+R16/17 (n=15) |
| --- | --- | --- | --- |
| Age (mo) | 6.5±1.3 | 6.9±0.7 | 5.7±0.6 |
| BW (g) | 24.1±1.3 | 29.6±1.7 | 28.3±0.6 |
| Left TW (mg) | 60.6±3.6 | 39.8±1.8 | 41.4±1.6 |
| Right TW (mg) | 56.6±3.6 | 37.1±1.9 | 39.8±1.4 |
| Left LW (mg) | 1375.0±68.3 | 1223.0±37.6 | 1318.0±27.0 |
| Right LW (mg) | 1392.0±83.5 | 1218.0±45.3 | 1233.0±34.0 |
| Left KW (mg) | 179.0±12.0 | 196.0±9.0 | 202.0±5.9 |
| Right KW (mg) | 178.0±12.0 | 205.0±9.0 | 217.0±5.5 |
| Ratio of Kidney MD L/R | 0.92±0.05 | 1.02±0.04 | 1.04±0.04 |

mo: Month; BW: Body Weight; TW: TA Muscle Weight; LW: Hindlimb Weight; KW: Kidney Weight; MD: microsphere distribution.
